# Quantitative assessment of cell fate decision between autophagy and apoptosis

**DOI:** 10.1038/s41598-017-18001-w

**Published:** 2017-12-14

**Authors:** Bing Liu, Zoltán N. Oltvai, Hülya Bayır, Gary A. Silverman, Stephen C. Pak, David H. Perlmutter, Ivet Bahar

**Affiliations:** 1 Department of Computational and Systems Biology, University of Pittsburgh, Pittsburgh, PA 15213 USA; 2 Department of Pathology, University of Pittsburgh, Pittsburgh, PA 15261 USA; 3 Department of Critical Care Medicine, University of Pittsburgh, Pittsburgh, 15213 PA USA; 4 Department of Pediatrics, Washington University School of Medicine, St Louis, Missouri, MO 63110 USA

## Abstract

Autophagy and apoptosis are cellular processes that regulate cell survival and death, the former by eliminating dysfunctional components in the cell, the latter by programmed cell death. Stress signals can induce either process, and it is unclear how cells ‘assess’ cellular damage and make a ‘life’ or ‘death’ decision upon activating autophagy or apoptosis. A computational model of coupled apoptosis and autophagy is built here to analyze the underlying signaling and regulatory network dynamics. The model explains the experimentally observed differential deployment of autophagy and apoptosis in response to various stress signals. Autophagic response dominates at low-to-moderate stress; whereas the response shifts from autophagy (graded activation) to apoptosis (switch-like activation) with increasing stress intensity. The model reveals that cytoplasmic Ca^2+^ acts as a rheostat that fine-tunes autophagic and apoptotic responses. A G-protein signaling-mediated feedback loop maintains cytoplasmic Ca^2+^ level, which in turn governs autophagic response through an AMP-activated protein kinase (AMPK)-mediated feedforward loop. Ca^2+^/calmodulin-dependent kinase kinase β (CaMKKβ) emerges as a determinant of the competing roles of cytoplasmic Ca^2+^ in autophagy regulation. The study demonstrates that the proposed model can be advantageously used for interrogating cell regulation events and developing pharmacological strategies for modulating cell decisions.

## Introduction

Autophagy is a cytoprotective homeostatic process in which cells digest their own cytoplasmic constituents or organelles, and degrade them in the lysosomes, in response to diverse stress stimuli^1^. The resulting products can be recycled to generate energy and build new proteins, hence the activation of autophagy as a protective mechanism against starvation^2^. Autophagy also serves as a cellular quality control process that removes damaged organelles or aggregates of misfolded proteins that may otherwise cause a broad range of diseases, including neurodegenerative disorders^3^ and liver diseases^4,5^. Yet, excessive autophagy has been linked to ‘autophagic’ cell death, and autophagy activation has been pointed out to be harmful under certain disease conditions (e.g. cancer)^6^, while recent studies suggest that autophagy might represent in those cases an attempt to prevent the inevitable demise of the dying cells^7^. The modulation of autophagy has thus emerged as an important therapeutic strategy for several diseases^8,9^.

Due to a complex crosstalk between autophagy and apoptosis^6^, it is often unclear which specific interactions contribute to pro-survival or pro-death effects in a given disease. A database has been developed^10^ for mining the network of protein-protein interactions as well as transcription factors and miRNAs implicated in autophagy regulation. While this database is a valuable resource that provides information on autophagy components and regulators, there is a need to build in parallel models and methods that can leverage existing data and assist in making mechanistic inferences on the dynamics of autophagic interactions. Our goal here is to present such a tractable mathematical model to assess how cells orchestrate the dynamics of signaling networks to make ‘life’ vs. ‘death’ decisions, and how these are modulated by pharmacological interventions.

Our model includes mTOR and inositol signaling autophagic pathways and intrinsic apoptosis pathways as well as their crosstalks mediated by Bcl2, caspases, p53, calpain and Ca^2+^. As will be shown below, using a statistical model checking (SMC)-based framework^11^, we generated a calibrated model that captures cellular heterogeneity, and closely reproduces the differential initiation and time evolution of autophagy or apoptosis in response to nutritional, genotoxic, or endoplasmic reticulum (ER) stresses observed in single-cell experiments^12^.

The model points to AMP-activated protein kinase (AMPK) as a key mediator of the competing roles of intracellular (IC) Ca^2+^, designated as Ca^2+^(IC). The Ca^2+^(IC) level, [Ca^2+^(IC)], acts as a rheostat that fine-tunes autophagic and apoptotic responses, regulated by a positive (G-protein signaling) feedback loop and Ca^2+^/calmodulin-dependent kinase kinase β (CaMKKβ) level. The model also enables the rapid assessment of the effect of a series of drugs on the onset and development of autophagy or apoptosis, under different stress conditions.

## Results

### Quantitative model of coupled autophagy and apoptosis signaling network

**Figure 1:**
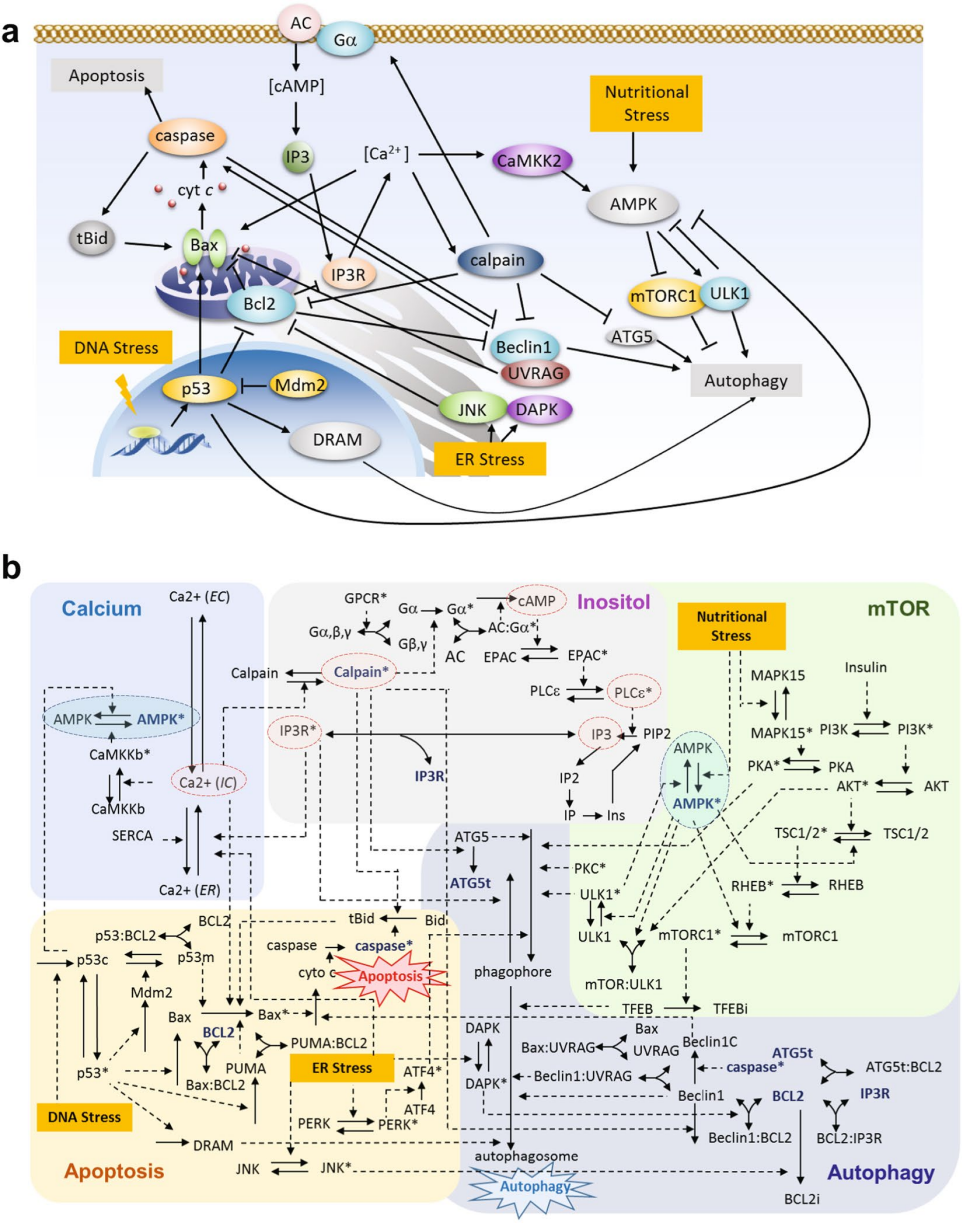
Reaction network model for autophagy-apoptosis crosstalk. (**a**) Schematic illustration of the main components and their key interactions. Activating and inhibitory interactions are distinguished by different types of arrows. Full names of compounds are given in Supplementary Table S1. (**b**) A more detailed diagram depicting the network of protein-protein and protein-ion/metabolite interactions. The network is composed of five coupled modules (calcium, inositol, mTOR, apoptosis and autophagy), shown in different background colors. *Solid* and *dashed* arrows refer to physical (association/disassociation/translocation) and chemical reactions, respectively. The complete list of reactions and interactions is presented in the Supplementary Table S2. Some components involved in multiple modules (e.g. AMPK, IP_3_R, Bcl-2, Bax, Atg5) are shown at multiple places, for clarity. Selected compounds/reactions identified as critical mediators of cell response are highlighted *in red/blue* ellipses.

The model is composed of five modules, which includes the major signaling cascades activated in response to nutritional, genotoxic, and ER stresses (Fig. 1a). The system is composed of 94 components, including the different activation, binding or localizations states of involved proteins, and the dynamics of this system is represented by a system of ordinary differential equations (ODEs). Supplementary Tables S1 and S2 list the components (and acronyms), rate equations and parameters. We present below a brief description of each of these five modules (Fig. 1b).

#### Apoptosis module

Following our previous model^13^, nuclear p53 gains transcriptional activity for pro-apoptotic proteins represented by Bax and its activator, PUMA, under genotoxic (DNA) stress. Activated p53 induces Mdm2, which, in turn, inhibits p53 by facilitating its ubiquitination and translocation to the mitochondrium.

Mitochondrial p53 inhibits the anti-apoptotic protein Bcl-2, and activates Bax to promote the formation of mitochondrial outer membrane permeability pores for the release of cytochrome *c* (cyt *c*), which leads to caspase activation. The process is amplified by a positive feedback loop in which caspase-truncated Bid (tBid) induces the activation and subsequent oligomerization of Bax.

ER stress also triggers apoptosis, for example, through activation of c-Jun N-terminal kinases (JNK)-, protein kinase R (PKR)-like ER kinase (PERK)-, and death-associated protein kinase 1 (DAPK1)-dependent pathways^14,15^. Further activation of caspase cascades by calpain due to the stress-induced release of ER Ca^2+^ to the cytoplasm is described in the calcium module.

#### Autophagy module

Autophagy involves the formation of a phagophore, which engulfs dysfunctional substrates, protein aggregates, or organelles to form autophagosomes. The content of the autophagosome is degraded through the lysosomal machinery^3^. Autophagic elimination involves several protein complexes such as the mTOR complex 1 (mTORC1; autophagy suppressor) and the Unc-51-like autophagy-activating kinase 1 (ULK1; autophagy promoter). Our model also includes mediators of autophagy progression such as Atg5, Beclin-1 and UV radiation-resistance associated gene (UVRAG) protein and regulatory proteins (e.g. PKA and PKC) that inhibit autophagy by phosphorylating LC3^16^.

The above two processes are coupled in multiple ways: (i) UVRAG inhibits Bax, while it interacts with Beclin-1 to promote autophagy^17^; (ii) Bcl-2 inhibits autophagy by interacting with Beclin-1^18^, and can be suppressed by truncated Atg5^19^; (iii) Bcl-2 also enhances autophagy via its interaction with inositol 1,4,5-trisphosphate receptor (IP_3_R), an inhibitor of autophagosome formation^20^; (iii) Activated caspases can cleave Beclin-1 to inhibit autophagy^21^ and C-terminal Beclin-1 fragments enhance apoptosis by promoting the release of cyt *c* from mitochondria^22^; (iv) Cytoplasmic p53 inhibits autophagy by deactivating AMPK^23^, while nuclear p53 promotes autophagy via transcriptional activation of damage-regulated autophagy modulator (DRAM) - a lysosomal protein that induces autophagy^24^, and stimulation of JNK signaling pathways to trigger Bcl-2 phosphorylation^25^; (v) ER stress also activates JNK, which phosphorylates (and inactivates) Bcl-2^26^; it also activates DAPK, which dissociates from Bcl-2:Beclin-1 complex^27^; (vi) Activated calpain cleaves Atg5 and Beclin-1 to inhibit autophagy, and truncates Bid to induce apoptosis^19,28^.

#### mTOR module

Under normal condition, mTORC1 is phosphorylated and active (designated with superscript*); mTORC1* binds ULK1 thus preventing its activation, and inactivates the transcription factor EB (TFEB)^8^, which are essential proteins promoting autophagy. Under cellular stress, stimulation of PI3K-AKT-TSC1/2-RHEB pathway inactivates mTORC1*, leading to the release of ULK1 and activation of autophagy^16^. In parallel, nutrient stress is sensed by AMPK, which inhibits mTORC1 pathways as a mechanism for suppressing cell growth and biosynthesis^29^. Specifically, AMPK releases and thus activates ULK1 which induces autophagy. It also inactivates mTORC1* by triggering the TSC1/2-RHEB cascade and directly phosphorylating a protein (Raptor) in mTORC1^16^. Furthermore, AMPK is negatively regulated by cytoplasmic p53 and ULK1* and positively regulated by CaMKKβ (see the calcium module)^30^.

#### Inositol module

The module is activated upon ligand-binding to G-protein coupled receptors (GPCRs), which prompts the dissociation of the α-subunit of the intracellularly bound G protein from the β- and γ-subunits. Dissociation of activated Gα*s* subtype, Gα*, stimulates the production of cyclic AMP (cAMP) upon binding onto and activating adenylate cyclase (AC) that catalyzes the conversion of ATP to cAMP. The effects of Gα subtypes Gαq and Gαi on AC are implicitly included through model parameters. cAMP blocks autophagy by activating the exchange protein EPAC which, in turn, activates the phospholipase Cε (PLCε). PLCε* induces the production of IP_3_ and consequently, the release of Ca^2+^ from ER upon binding of IP_3_ to its receptor IP_3_R, a ligand-gated Ca^2+^ channel, on the ER membrane^8^.

#### Calcium module

Ca^2+^ translocates between the extracellular (EC) space, the cytoplasm and the ER, regulated by voltage-gated and ligand-gated ion channels (e.g. IP_3_R) and pumps (e.g. SERCA)^31^. Ca^2+^(IC) activates CaMKKβ, which phosphorylates (or activates) AMPK to promote autophagy^32^ (see mTOR module). CaMKKβ also activates calpain. Calpain* activates the inositol pathway, inhibits autophagy (by cleaving Atg5)^19^, and/or induce apoptosis by activating Bax^33^.

### The calibrated model reproduces differential dynamics of autophagy and apoptosis in response to nutritional-, genotoxic-, and ER-stress

**Figure 2:**
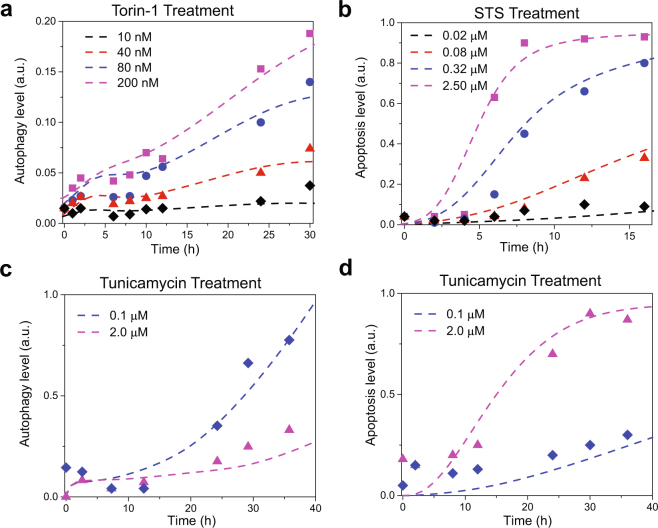
Comparison of model predictions with experimental training data. Experimental and simulated time evolution of autophagic and apoptotic response of H4 cells to Torin-1 (**a**), staurosporine (STS) treatment (**b**), and tunicamycin treatment (**c**,**d**) are shown. Dashed curves represent the results from simulations; the symbols designate the experimental data points extracted from Xu *et al*.^12^.

We utilized image-based single-cell experimental data^12^ generated in human neuroglioma H4 cells to estimate the unknown parameters. These cells express both the GFP-LC3 and histone cluster 2-RFP (H2B-RFP) reporters. The GFP-LC3 reporter delineates cell boundaries and also serves as an autophagy marker, while the H2B-RFP reporter was used as a marker of both nuclear boundary and apoptosis^12^. The training data for model calibration is the time courses of autophagy and apoptosis under: (i) 10, 40, 80, and 200 nM Torin 1 (or rapamycin, mTOR inhibitor) treatment (Fig. 2a), (ii) 0.02, 0.08, 0.32 and 2.5 μM staurosporine (STS, cytotoxic reagent that inhibits several kinases including PKC and PKA) treatment (Fig. 2b), and (iii) 0.1 and 2.0 μM tunicamycin (ER stress-inducer) (Fig. 2c,d). The resulting kinetic parameters are listed in Supplementary Table S2. Figure 2 displays the excellent agreement achieved between the profiles generated by our simulations (*dashed curves*) and the experimental data (*dots*), using the optimized set of parameters.

**Figure 3:**
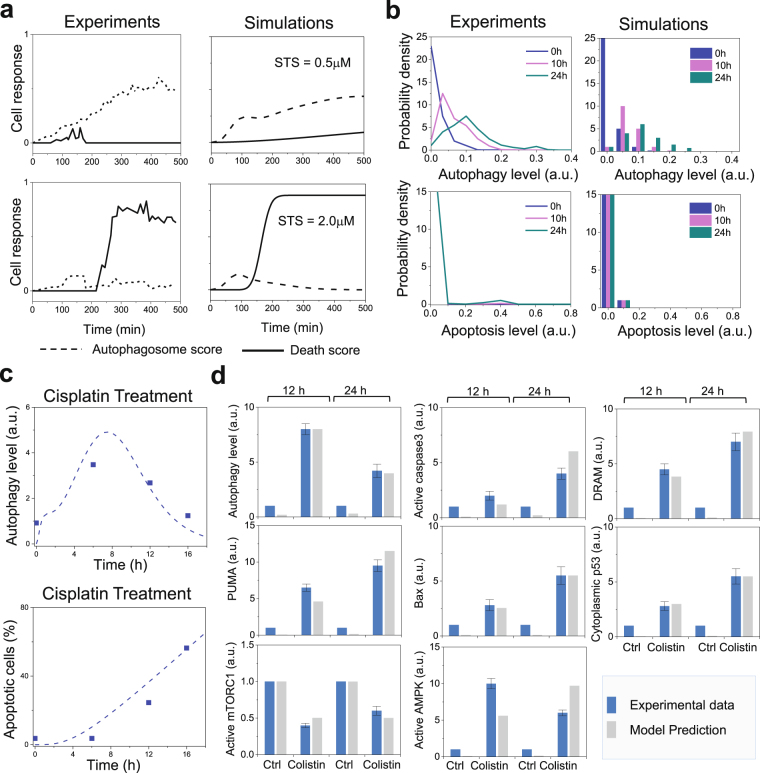
Validation of the integrated model upon comparison of predictions with independent data generated in different cell lines. (**a**) Experimental and simulated time evolution of autophagic and apoptotic responses of a single H4 cell to STS treatment. (**b**) Experimentally observed probability densities and model-predicted histograms of autophagy and apoptosis levels in a population of H4 cells in response to 0, 10 and 24 h of starvation. (**c**) Experimental and simulated time evolution of autophagic and apoptotic responses of RPTC cells to cisplatin treatment. The dashed curves are obtained by computations; symbols designate the experimental data points. (**d**) Experimental and simulated abundance of LC3-II, cleaved caspase 3, DRAM, PUMA, Bax, phosphorylated AMPK, and the active form of mTOR in PC-12 cells in response to 12 and 24 h colistin treatment. The experimental data in panels a,b,c and d, refer to the results from Xu *et al*.^12^, Periyasamy-Thandavan *et al*.^34^, and Zhang *et al*.^35^, respectively.

We next proceeded to the validation of our model. Comparison of experimental^12^ (*left panels;* adapted from Fig. 5A,B of Xu *et al*.^12^) and computational results (*right panel*) in Fig. 3a demonstrates that our computations reproduce the differential dynamics of autophagy and apoptosis observed in the single-cell analysis of H4 cells. Specifically, STS-induced stress can stimulate autophagy (*dotted curve*) and/or apoptosis (*solid curve*) in the same individual cells, and autophagy precedes apoptosis. Under low stress (0.5 μM STS*; top diagrams*), the onset of autophagy protects the cells from death. In contrast, under high-stress conditions (2.0 μM STS; *bottom diagrams*), temporary activation of autophagy (near *t* = 100 min) is not sufficient to prevent apoptosis: the early autophagic response disappears with the cell’s commitment to apoptosis.

Figure 3b shows the comparison of the model-predicted histograms (*right panels*) with the experimentally observed probability densities (*left panels*) of autophagy (*top*) and apoptosis (*bottom*) levels in a population of H4 cells in response to 0, 10 and 24 h of starvation. 1,000 trajectories were generated in line with the prior distributions of initial concentrations (see *Methods*). The simulations accurately reproduce the experimentally observed induction of autophagy (and not apoptosis) upon inhibition of mTOR. The distributions predicted in autophagy levels of the cells under the same starvation conditions, consistent with experimental observations, indicate that the model captures cell-to-cell variability, in addition to the average behavior.

We further validated our model using two additional datasets generated in rat kidney proximal tubular (RPTC) cells^34^ and rat adrenal medulla PC-12 cells^35^, respectively. Figure 3c shows the corresponding model predictions (*dashed curves*), which quantitatively reproduce the experimentally observed (*dots*) time courses of autophagy and apoptosis of RPTC cells in response to 20 μM cisplatin (ER stress-inducer). Here the autophagy level was measured as the densitometry of LC3-II signals in immunoblots; and the apoptosis level was reported as the percentage of apoptotic cells assessed by morphological methods^34^. Figure 3d further shows that the model predictions (*gray bars*) are consistent with the western blotting-based experimental data (*blue bars*): mainly, 12 and 24 h treatments with 125 μg/ml colistin (genotoxic stress-inducer) increase the abundance of LC3-II (autophagy marker), activated caspase 3, DRAM, PUMA, Bax, phosphorylated AMPK, and cytoplasmic p53, and have an inhibitory effect on the expression of the activated form of mTOR in PC-12 cells.

The above results show that our model predictions quantitatively match not only the training data (H4 cells, Fig. 2) but also the independent test data (H4, RPTC, and PC-12 cells, Fig. 3). The slight difference between the predicted and measured AMPK* level may be due to the simplifications assumed by our model and further refinement is probably necessary. The high predictive-performance of our model benchmarked against these diverse testing data establishes the validity of the model and confirms that the model parameters have not been overfitted. With that, we now proceed to further investigating and unravelling the major effects and mechanisms that regulate autophagy/apoptosis under different stress conditions.

### Sensitivity analysis indicates that Ca^2+^ release from ER, and regulation by p53, calpain, AMPK are key determinants of cell fate

**Figure 4:**
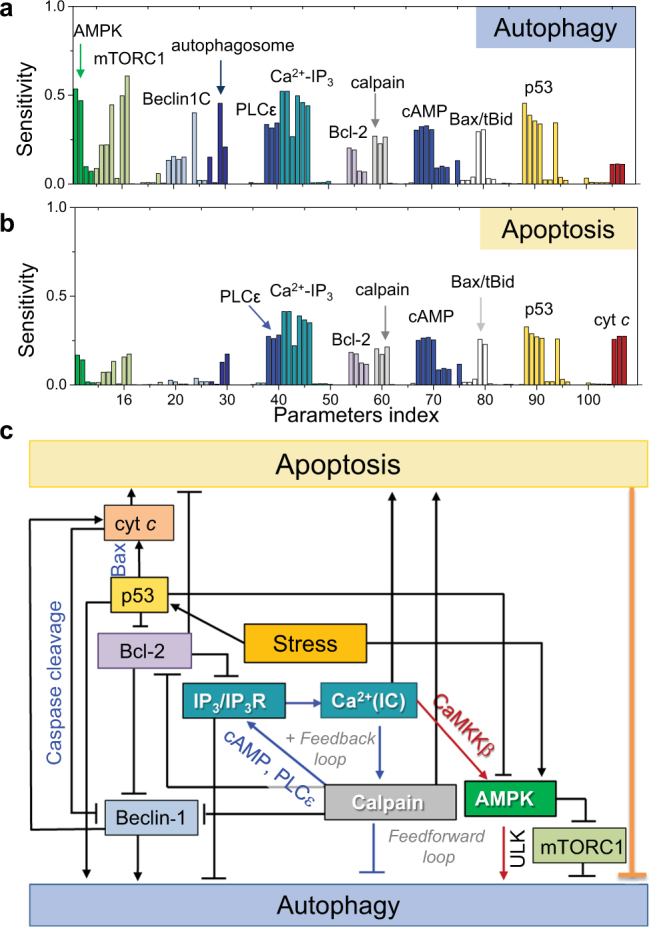
Sensitivity analysis. Peaks display the reactions/interactions distinguished by their strong effect on autophagy (**a**) and apoptosis (**b**), grouped by the corresponding major component (*labeled*). Parameter index (abscissa) refers to Supplementary Table S2. (**c**) The core regulatory network composed of key determinants of cell decision.

We first made a quantitative assessment of the components and reactions that are essential to cell fate decision. To this aim, we used a multi-parametric sensitivity analysis (MPSA) based on SMC^11^ (see *Methods*). Outputs used as criteria were autophagy and apoptosis levels induced by 0.5 μM STS. Figure 4 panels a,b present the global sensitivity values to various reactions, organized by representative reactants (labeled in different colors), obtained by varying the kinetic parameters within the ranges listed in the Supplementary Table S3.

Components whose kinetics exert strong effects on cell regulation/death are classified into three clusters: those influential on (i) both autophagy and apoptosis, (ii) only autophagy, and (iii) only apoptosis. The inositol module reactions associated with cAMP-PLCε-IP_3_-IP_3_R*-Ca^2+^-calpain- pathway (highlighted in *red ellipses* in Fig. 1b) emerge as major determinants of both autophagy and apoptosis. This points to the role of [Ca^2+^(IC)] released from the ER as the product of this pathway, which further promotes this pathway as an activator of calpain.

p53 also plays an important role in both apoptosis and autophagy, and couples to Ca^2+^ signaling via Bcl-2. Both Ca^2+^ and p53 regulate the activation/inhibition of AMPK (*dark blue ellipses* in Fig. 1b), which, in turn, favors autophagy, hence the emergence of the AMPK peak in Fig. 4a.

**Figure 5:**
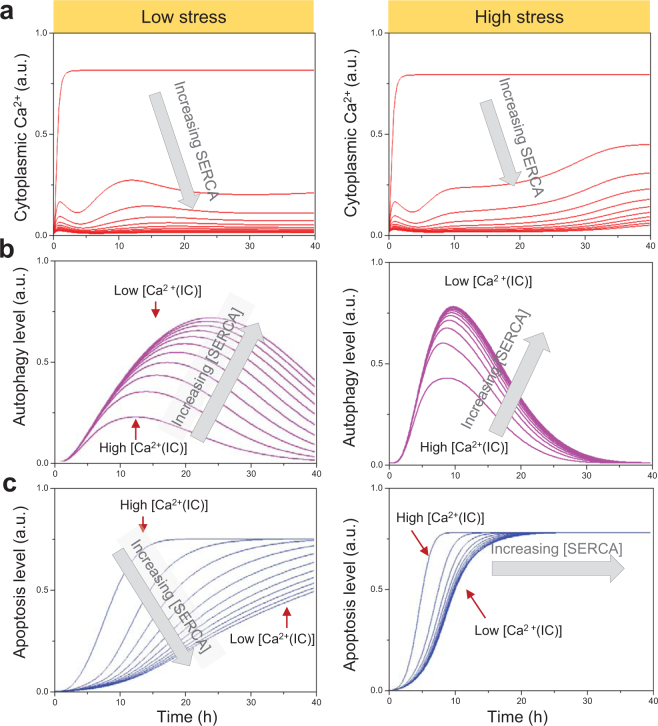
Role of [Ca^2+^(IC)] in the onset of autophagy vs apoptosis as a function of stress intensity. **(a)** Time evolution of [Ca^2+^(IC)] as [SERCA] varies from to 1 to 100 nM for low dose (0.5 μM) of STS and high dose (2 μM) of STS. **(b,c)** Accompanying time evolutions of autophagy **(b)** and apoptosis **(c)**.

Beclin-1, like calpain, p53 and AMPK, occupies a central role in the crosstalk between the two pathways. Notably, the cleavage of Beclin-1 by caspases inhibits autophagy, and contributes to the overall commitment to apoptosis via a positive feedback loop that promotes cyt *c* release (Fig. 1b). Protease-induced changes of autophagy proteins such as Beclin-1, Atg5 have been reported to trigger a switch from autophagic to apoptotic response^19,22,28^. In our model, the autophagy-inhibitory effect of Beclin-1 cleavage is apparent by the sharp (*light blue*) peak in Fig. 4a, while the apoptosis-enhancing effect is eclipsed by that of Bax/tBid-mediated apoptosis-amplifying loop.

The peaks appearing in both panels a and b of Fig. 4 thus underscore the biphasic behavior of autophagy observed in H4 cells in response to increasing STS dosage^12^. The cell’s first homeostatic response appears to induce autophagy at low stress/toxicity but it resorts to apoptosis with increasing stress levels. The onset of apoptosis is accompanied by termination of autophagy, hence the observed modest control of autophagy by components known to dominate apoptosis.

A schematic description of the flow of information between the key components distinguished in the above sensitivity analysis is presented in Fig. 4c. At the center of the diagram is the release of Ca^2+^ by IP_3_R fueled by a positive feedback loop (*blue arrows*) involving calpain*, cAMP, and PLCε*. The net effect of this loop is to suppress autophagy. However, this effect is countered by Bcl-2 that inhibits the IP_3_R. Yet, Bcl-2 simultaneously suppresses autophagy by inhibiting Beclin-1; whereas calpain* and p53 attenuate the effects of Bcl-2. The diagram also points to two competing roles of increased [Ca^2+^(IC)]: suppression of autophagy through calpain*; and promotion of autophagy, via a feedforward loop that involves CaMKKβ* and AMPK*. The autophagy-upregulating role of AMPK* is further reinforced by inhibition of mTORC1 and upregulaton of ULK1; however, AMPK* is deactivated by p53. Thus, ER membrane Ca^2+^ channels (represented here by IP_3_R), p53, AMPK, calpain, and Bcl-2 emerge as master regulators of cell decision between apoptosis and autophagy, their effect being closely associated with the modulation of intracellular Ca^2+^ levels.

### Cytoplasmic Ca^2+^ functions as a rheostat that fine-tunes the timing of autophagic and apoptotic responses

We next turn our attention to the mechanism of action of Ca^2+^(IC). The release of Ca^2+^ from the ER is enabled upon activation of ER membrane receptors by secondary messengers (i.e. IP_3_R activation by IP_3_). Ca^2+^ is conversely transferred from the cytoplasm to the ER lumen by ATPase pumps such as sarco/ER Ca^2+^-ATPase (SERCA). To investigate the effect of alterations in [Ca^2+^(IC)] on autophagy regulation, we increased *in silico* the expression level of SERCA and simulated the autophagy profiles in response to low and high levels of stresses (Fig. 5). Note that the same effect on [Ca^2+^(IC)] could alternatively be induced by inhibiting Ca^2+^ channels on cell membrane or ER membrane.

Figure 5a shows the decrease in [Ca^2+^(IC)] upon increasing [SERCA]_0_ at low (*left*) and high (*right*) stress. At low stress, a graded increase in autophagic response is observed *in silico* (Fig. 5b); whereas under high stress, the autophagic response is faster and of shorter duration: it reaches its peak around 10 h, even with moderate increases in [SERCA]_0_, after which it gives way to apoptosis (Fig. 5c), i.e. the onset of apoptosis concurs with the weakening of autophagy. A surge in apoptotic response is robustly elicited by the initial reduction in [Ca^2+^(IC)] in accordance with the bistability of apoptosis^36^, the transition being sharper and faster under high stress. Decrease in [Ca^2+^(IC)] under low stress *in silico*, on the other hand, exerts a moderate effect on the strength and/or timing of apoptotic response (Fig. 5b).

Taken together, our *in silico* results suggest a ‘rheostat’ mechanism regulated by calcium signaling, which enables the cell to fine tune its response to stress. The cell copes with low stress conditions by initiating an autophagic response, if [Ca^2+^(IC)] is sufficiently low; but if [Ca^2+^(IC)] is high, the same mediators of cell response that otherwise favor autophagy alter the cell commitment toward programmed death, the switch in the behavior being sharper and faster under high stress.

### Gα signaling and ensuing PLCε activation maintain cytoplasmic Ca^2+^ level through a positive feedback loop

As illustrated in Fig. 1a and summarized in Fig. 4c, [Ca^2+^(IC)] is regulated by a positive feedback loop formed by the G-protein α-subunit that drives cAMP production by AC, and IP_3_ signaling that in turn activates calpain, which further stimulates the inositol pathway, and so on. Here we focus on this effect. To this aim, we varied the initial concentration, [AC]_0_, of AC, and evaluated the effect on cAMP production and ensuing stimulation of EPAC and PLCε on [Ca^2+^(IC)]. We also repeated the simulations under high and low influx of Ca^2+^ to the cytoplasm from other sources, here modulated by varying SERCA levels/activity.

**Figure 6:**
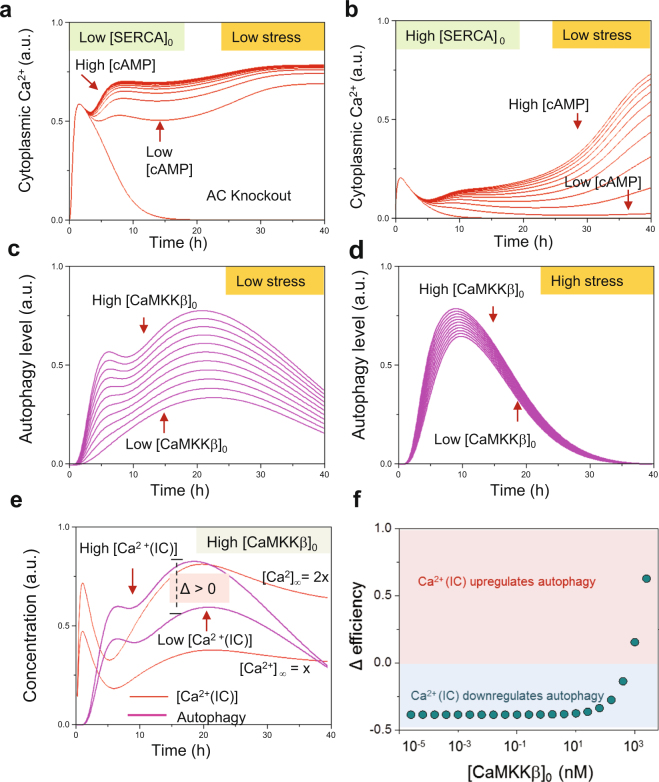
Significance of cAMP and CaMKKβ in modulating the response of the cell to varying IC Ca^2+^ levels. The *in silico* cellular stress is induced by administering a low dose (0.5 µM) of STS, except for panel **f** where [STS] = 2 µM. (**a,b**) Time evolution of [Ca^2+^(IC)] under different initial concentrations of AC, for low (**a**) and high (**b**) [SERCA]. [cAMP] produced by AC varies from 10 (low) to 100 nM (high). (**c,d**) Simulated development of autophagy for 0.01 < [CaMKKβ]_0_ < 1 nM, under low (**c**) and high (**d**) stress. The propensity of the cell for autophagy increases with increase in [CaMKKβ]_0_. (**e**) Simulated profiles of autophagy (*magenta*) accompanying the changes in [Ca^2+^(IC)] (*red*) in the presence of elevated [CaMKKβ]_0_. The enhancement in autophagy level due to change in [Ca^2+^(IC)]_∞_ by a factor of 2, is designated by Δ, which is the maximum difference between the two curves. (**f**) Dose-response curve of Δ as a function of [CaMKKβ]_0_. IC Ca^2+^ downregulates autophagy in general (see Fig. 5) despite the opposing effect of CaMKKβ, except for elevated (>10^3^ nM) [CaMKKβ].

Figurementary Figure S1a shows that knocking down AC leads to a graded reduction in cAMP level as expected, the reduction being more pronounced with high [SERCA]_0_ (100 nM). Of interest is the concurrent non-uniform changes in [Ca^2+^(IC)] (Fig. 6a). [Ca^2+^(IC)] exhibits a complex time evolution, depending on the extent of downregulation of cAMP (or EPAC/PLCε) and upregulation of SERCA: (i) when the supply of Ca^2+^ is sufficiently high (e.g. with [SERCA]_0_ = 10 nM), [Ca^2+^(IC)] is bistable; it maintains a high level even with a small stimulation of EPAC/PLCε activation via G-protein signaling, but it is severely depleted in the absence of such signaling (Fig. 6a), (ii) in the opposite case of an upregulated SERCA which promotes the removal of Ca^2+^(IC) from the cytoplasm into the ER, there is a first decrease in [Ca^2+^(IC)]; but the suppression of [Ca^2+^(IC)] cannot be sustained due to the restoring effect Gα-signaling after 24 h, approximately. (Fig. 6b). The positive feedback loop mediated by calpain and PLCε thus plays a key role in restoring and maintaining the physiological cytoplasmic Ca^2+^ levels. Once Ca^2+^(IC) is boosted to a certain level, it robustly maintains its level by this cellular feedback.

### CaMKKβ shapes the role of cytoplasmic Ca^2+^ in regulating autophagy

Cytoplasmic Ca^2+^ plays a dual role in autophagy: it inhibits autophagy upon activation of calpain and IP_3_R; and enhances autophagy upon activation of AMPK in a CaMKKβ-dependent manner (respective *blue* and *red arrows* in Fig. 4c). This forms an incoherent type 1 feedforward loop (I1-FFL), a motif frequently seen in biological networks^37^. The effect of cytoplasmic Ca^2+^ thus depends on the balance between these opposing actions. Which effect dominates, under which conditions? Fig. 5 suggests the dominance of the former (at least in H4 cells under low stress, with [SERCA]_0_ varying in the range 1–100 nM), that is, increase in [Ca^2+^(IC)] suppresses autophagy and promotes apoptosis.

Given the complexity of [Ca^2+^(IC)] time evolution observed in Fig. 6a,b, we investigated whether there might be a reversal in the anti-autophagic/pro-apoptotic effect of Ca^2+^(IC) increase upon modifying the levels/rates of other components/reactions in the calcium signaling module. To this aim, we increased the amount of activated CaMKKβ. Figure 6c obtained under the same conditions with [SERCA]_0_ = 77 nM, confirms that increased [CaMKKβ]_0_, which also leads to higher [CaMKKβ*], enhances autophagy. This effect is, however, temporary; it disappears after 20–40 hours, as autophagic response gives way to apoptosis. The termination of autophagy is expedited (with minimal dependence on [CaMKKβ]) under high stress (Fig. 6d).

To evaluate the overall role of cytoplasmic Ca^2+^ in regulating autophagy, we define a variable Δ that measures the *change in autophagy level* in response to doubling the steady state level [Ca^2+^(IC)]_∞_ of cytoplasmic Ca^2+^. As shown in Fig. 5e, for high [CaMKKβ]_0_, doubling [Ca^2+^(IC)]_∞_ (e.g. by knocking down SERCA) enhances autophagy (Δ > 0). In contrast, for low [CaMKKβ]_0_, doubling [Ca^2+^(IC)]_∞_ results in a decrease in autophagy (Δ < 0) (Supplementary Fig. S2).

This analysis implies that whether cytoplasmic Ca^2+^ up- or down-regulates autophagy depends on the initial concentration of CaMKKβ. Figure 6f shows the response curve of Δ as a function of [CaMKKβ]_0_. While the effect of increased Ca^2+^ levels remains proapoptotic for a broad range of [CaMKKβ]_0,_ beyond a certain (high) level, there is a switch to pro-autophagic response. This result suggests that cell types with different levels of CaMKKβ expression may exhibit opposite responses to autophagy-modulating drugs (e.g. verapamil) that target [Ca^2+^(IC)].

### *In silico* simulations reveal potential pharmacological strategies for controlling cell fate

**Figure 7:**
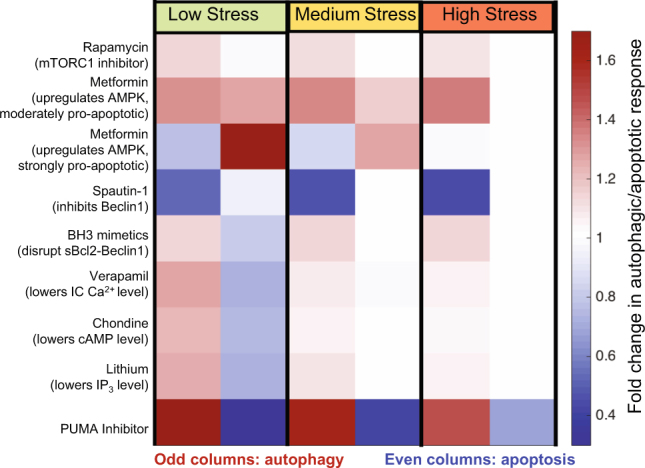
Cell response to different treatments predicted *in silico* under different stress conditions. The simulated autophagy and apoptosis level in response to low, medium, and high dose of STS stress, when the cells are subjected to the drugs listed along the left abscissa. The color-coded entries represent the fold change in autophagic (odd columns) and apoptotic (even columns) responses, relative to those in the absence of treatment.

The model developed here permits us to interrogate various treatment scenarios and evaluate their efficacies for either enhancing autophagy to protect normal cells or enhancing apoptosis to kill cancer cells. Figure 7 illustrates the simulated treatment efficacy of low dose of eight FDA-approved drugs under three different levels of stress. The known (major) target and action of each drug are indicated along the left abscissa in Fig. 7. For each level of stress, the left column shows the extent of autophagic response, and the right column shows the extent of apoptotic response, i.e. odd and even columns represent the predicted fold changes in autophagy and apoptosis levels, respectively, in response to drug treatment. In general enhancement of autophagy is accompanied by suppression of apoptosis and *vice versa*, although the individual drugs, under different stress conditions exhibit rather complex effects due to the involvement of their targets in multiple interconnected pathways that may have counter effects, as we discuss below.

The mTORC1 inhibitor rapamycin enhances autophagy irrespective of the stress level, and has practically no effect on apoptosis. This is consistent with experimental observations using H4 cell line (neuroglioma)^38^.

AMPK was distinguished here as a key up-regulator of autophagy, and upregulating AMPK enhances autophagy and may slightly hinders apoptosis due the elimination of the stress by autophagy (Supplementary Fig. S3). Metformin is known to upregulate AMPK, while a recent study also indicate that metformin downregulates the expression of Bcl-2 and upregulates the expression of Bax^39^. Inclusion of these promiscuous effects of metformin in the model, led to either simultaneous enhancement of autophagy and apoptosis or enhanced apoptosis but suppressed autophagy, depending on the strength of pro-apoptotic effects of metformin on a particular cell line (Fig. 7). These results are consistent with the observations that metformin promote autophagy and apoptosis in esophageal squamous cell carcinoma^40^, while promoting apoptosis but suppressing autophagy in glucose-deprived H4IIE hepatocellular carcinoma cells^41^. The model thus permits us to better assess the effects of promiscuous drugs, or interpret their polypharmacological effects.

Spautin-1, an inhibitor of Beclin-1, effectively suppresses autophagy under all conditions and its effect on apoptosis is minimal, except for low stress conditions. This is consistent with the experimental observations in H4 cells and Madin-Darby canine kidney epithelia (MDCK) cells^42^.

BH3 mimetics (which disrupts the interaction between Bcl-2 and Beclin-1), verapamil (which lowers [Ca^2+^(IC)]), clonidine (which lowers [cAMP]), and lithium (which lowers [IP_3_]) have similar actions and efficacies. Under low level of stress, they enhance autophagy and suppress apoptosis, while for medium and high levels of stress, they essentially enhance autophagy. BH3 mimetics (e.g. ABT-737) have been reported to induce autophagy for multiple cell lines (e.g. HeLa, U2OS, HCT116)^43,44^, suggesting that the interaction between Bcl-2 and Beclin-1 might be a robust drug target for enhancing autophagy. Both verapamil and clonidine have been shown to induce autophagy in PC12 cells^45^. Our prediction is also consistent with the observation that with the same dosage (1 µM), verapamil induces a higher level of autophagy than does clonidine. The role of lithium on inducing autophagy has been demonstrated in H4 cells and PC12 cells^46,47^.

Interestingly, PUMA inhibition shows remarkable pro-survival effects: it enhances autophagy and suppresses apoptosis under all conditions. This is consistent with the results from sensitivity analysis which highlighted the strong inhibitory effect of apoptosis on autophagy (via the cleavage of Beclin-1 by caspases). It implies that radiation mitigators may enhance pro-survival autophagy in injured cells to further prevent cell death. PUMA inhibitors have been shown to efficiently inhibit apoptosis using multiple cell lines^48^, while their upregulation of autophagy requires further confirmation.

Taken together, these results indicate that: (i) to protect normal cells against stress-induced cell death, upregulation of AMPK, which simultaneously activates multiple downstream autophagic pathways, and inhibition of PUMA^48^, could be highly efficacious strategies; (ii) In contrast, Beclin-1 inhibitors such as spautin-1 may improve the efficacy of apoptosis-inducers and may be advantageously used for pre-empting autophagic response and enabling the elimination of cancer cells with high apoptotic thresholds.

## Discussion

Autophagy has been referred to as a form of programmed cell death in several studies, named as ‘autophagic cell death’ (ACD) or ‘type II cell-death’, since programmed cell death was often associated with enhanced autophagy and depended on autophagy-associated proteins to a certain context^49^. However, recent evidence led to a debate on the existence of ACD^7,50^. Our current understanding is that though ACD exists in rare situations, a more general scenario is that autophagy precedes apoptosis in the same cell in order to adapt to or cope with non-lethal stress^1^, and apoptosis occurs when the stress exceeds a critical threshold of intensity or duration^6^. The present study provides firm quantitative description in support of the validity of this interplay between autophagy and apoptosis. In most cases, autophagy and apoptosis mutually inhibit each other and the onsets of autophagy and apoptosis are governed by cell fate decision processes that have broad pathophysiological implications^6,51^. We presented here a calibrated computational model (Fig. 1) for the kinetics of autophagic and apoptotic pathways crosstalk, which successfully reproduces a wealth of experimental data (Figs 2 and 3 **)**, and provides a computing platform for generating new hypotheses, including the predictions presented in Figs 4–7.

Our analysis identified a core regulatory network for autophagic and apoptotic responses (Fig. 4c). Specifically, cytoplasmic Ca^2+^, p53, AMPK, calpain, Beclin-1, and Bcl-2 emerged as key components whose expression and/or activities significantly affect cell decision. Ca^2+^ and p53 act as master regulators that tightly control cell decisions through AMPK and Bax activation pathways, respectively. Cleavage of Beclin-1 by caspases confers a concrete switch from autophagy to apoptosis. A positive feedback loop formed by components in the Gα signaling and inositol pathways including calpain is critical to exerting the pro-apoptotic effect of [Ca^2+^(IC)], which in parallel maintains a graded autophagic response through a feedforward loop mediated by CaMKKβ and AMPK. Our model also predicts that the CaMKKβ activation may act as a determinant of the dual/opposite roles of cytoplasmic Ca^2+^ as well as treatment efficacy. While increased [Ca^2+^(IC)] usually inhibits autophagy (in favor of apoptosis), this effect can be reversed in cells that express high levels of CaMKKβ.

Previous modeling works have focused on apoptosis^52–54^. Recent progress on modeling of autophagy either focused on specific modules such as autophagic vesicle dynamics^55^ and mTORC1-ULK1 interaction^56^ or used over-simplified networks^57,58^. In a recent study^59^, a relatively larger cancer-specific model of 13 ODEs has been built and calibrated. Unfortunately, the model has not been further advanced and thus no new knowledge has been derived so far from that model.

Here, we have developed a first comprehensive and calibrated kinetic model of the autophagy-apoptosis crosstalk. Our model consists of 94 components which cover many important stress-sensing pathways (Fig. 1b). The model is generic, and its parameters can be calibrated to capture the pathway activation/dynamics in specific cell types based e.g., on single-cell dynamical protein expression profiles. We have demonstrated that our model was able to reproduce single-cell and population-based measurements of autophagic and apoptotic responses of human neuroglioma cells, rat kidney proximal tubular cells, and rat adrenal medulla cells under nutritional, genotoxic, and ER stresses (Figs 2 and 3 **)**. LC3 is a widely used marker for autophagosome formation. However, both GFP-LC3 and LC3-II suffer from limitations as measures of autophagy level^60^. Fluorescence microscopy based GFP-LC3 approach also suffers from the difficulties of quantifying puncta number and distinguishing GFP-LC3 aggregates from true autophagosomes, while LC3-II measured in biochemical assays presumably include some population of LC3-II generated in an autophagosome-independent manner. To overcome these limitations, we have validated our model using both GFP-LC3 (Fig. 3a,b) and LC3-II (Fig. 3c,d) data. We have demonstrated that our calibrated model serves as a platform for gaining insights into the underlying time-dependent interactions (Figs 4–6 **)**, as well as interrogating the network of interactions and cell fate decisions toward determining treatment strategies in favor of autophagy (for healthy cells under stress) or apoptosis (for cancer cells) (Fig. 6 **)**.

Live imaging and single-cell analysis have indicated that autophagy proceeds via a graded dynamics whereas apoptosis onset obeys a switch-like behavior^12^. However, the underlying mechanism remained unclear. Our previous work^13^ has shown that the positive feedback loop, Bax* → caspase → tBid → Bax*, is critical to sustaining caspase activity, and thereby controlling the switch-like dynamics of apoptosis. This positive feedback loop is also observed here to be responsible for the all-or-none bimodal dynamics of apoptosis in the presence of the coupling to autophagic pathways, as it mediates the pro-apoptotic effects associated with other components such as calpain and Ca^2+^(IC). As autophagy precedes apoptosis, it could start eliminating dysfunctional entities when the stress levels are not sufficiently high to trigger the caspase/tBid feedback loop. Autophagic events thus delay, if not prevent, apoptosis (maintaining ‘off’ state). This points to the importance of intervention timing for pre-empting the potential commitment of the cell to apoptosis, while the cell is disposing dysfunctional elements via autophagy.

When the stress level or duration reaches an apoptotic threshold, the positive feedback loop that ensures the sustained caspase cascade and the apoptotic machinery switches from ‘off’ to ‘on’ state. Upon committing to apoptosis, the cell shuts down autophagy and recruits other proteins (otherwise involved in both autophagic and apoptotic events) to promote apoptosis. Previous work^57^ hypothesized that this change in course is mainly due to the cleavage of Beclin-1 by caspase. Our sensitivity analysis corroborates that Beclin-1 cleavage by caspases is of utmost importance (Fig. 4a). However, our sensitivity analysis also highlights other important players and interactions. In particular, a unique incoherent type 1 feedforward loop (I1-FFL) involving CaMKKβ, AMPK, calpain, cAMP and IP_3_R emerged here as a determinant of [Ca^2+^(IC)], and consequently up- or down-regulation of autophagy. A dual role of Ca^2+^(IC) in autophagy emerges from a large number of contradicting evidences^31^. Elevated levels of Ca^2+^ have been reported to promote autophagy, while inhibitors of intracellular Ca^2+^ currents have been also found to promote autophagy^61^. Our results indicate that the balance between the two (*red* and *blue*) branches in Fig. 4c defines the decision/fate of the cell. Calpain is part of the cAMP-mediated positive feedback loop for sustaining [Ca^2+^(IC)]; it also activates apoptosis via Bid activation. Thus, its complex role hinders calpain as a modulator to shape the role of Ca^2+^(IC). Controlling CaMKKβ levels on the other hand emerges as a viable therapeutic strategy.

CaMKKβ is a versatile regulator of the CaMKs and involved in regulating many cellular processes such as glucose homeostasis and inflammation^32^. The expression of CaMKKβ varies in different cell types and tissues. Our results imply that overexpression of CaMKKβ can switch cytoplasmic Ca^2+^ from an inhibitor to an enhancer of autophagy (Fig. 6f). Ca^2+^(IC)-modulating drugs (e.g. verapamil) should thus be used in a cell/context-dependent manner, since the expression and activity level of CaMKKβ might influence the drug’s efficacy. Recent evidence indicates that CaMKKβ is expressed at very low levels in normal prostate, but accumulates in prostate cancer cells^62^. Consequently, enhanced activation of the CaMKKβ-AMPK pathway may increase autophagy and elevate the threshold for the onset of apoptosis, and thereby prevent the elimination of prostate cancer cells. In such context, Ca^2+^(IC)-reducing drugs might be potentially used in combination with apoptosis-inducers to suppress autophagy and enhance apoptosis.

Calcium is released from the ER by ryanodine receptors, too^63^. The opening of ryanodine receptors is triggered by ADP ribose. This mechanism is similar to the IP_3_R-dependent calcium release, thus in our model, IP_3_R may be viewed as a membrane protein representative of ligand-gated receptors that release calcium from the ER, including RYR. Further, sources of Ca^2+^ intake from the EC region include voltage-gated calcium channels and ligand-gated receptors such as NMDA receptors and PMCA (and Na^+^/Ca^2+^ exchangers) that pump Ca^2+^ from the cytosol to the EC region^64^. In the current model, we implicitly modeled these processes by assuming constant level of Ca^2+^ intake from the EC medium, but these proteins actually represent alternative targets for modulating autophagic responses via altering Ca^2+^ levels. Extension of the current model to explicitly include these components and their interactions will increase the utility of the model as a platform for designing and evaluating alternative treatment strategies.

## Methods

### Mathematical modeling

The dynamics of the reaction network (Fig. 1b) was modeled as a system of 94 ODEs. The reactions and associated parameters are presented in Supplementary Table S2. Reaction rates for protein association, disassociation, catalysis, transcriptional regulation and decay were modeled using mass action kinetics. The cell-to-cell variability was addressed by assuming probability distributions over the initial concentration and rate constants (see Supplementary SI Methods for details). We defined the apoptosis score as a normalized value of [caspase*]. The integration of apoptosis score over time was then used to predict apoptotic cell percentage. We defined the autophagy score as a normalized value of [autophagosome]. *In silico* single-cell analysis (Fig. 3a) was performed by drawing a random sample from the distributions of initial states, while *in silico* population-based analysis was performed by drawing a representative set of samples (i.e. 1,000 samples for Fig. 3b, 100 samples for Figs 2, 3c,d, 5–7 **)** from the distributions of initial states. The ODE system was solved using SUNDAILS package^65^.

### Model calibration and sensitivity analysis

Parameter estimation and global sensitivity analysis were performed with statistical model checking (SMC)-based methods^11^ (see Supplementary SI Methods for details). We encoded the training data (Fig. 2) as a bounded linear temporal logic (BLTL) formula to construct an objective function and used our SMC tool to sample prior distributions of parameters and search for parameters with the global minimum objective values in the parameter space. The global sensitivity analysis was performed with a SMC-based MPSA method^11^. We encoded the training data on autophagy (Fig. 2a,c) and apoptosis levels (Fig. 2b,d) separately as BLTL formulae to construct two objective functions. A representative set of samples were drawn from the parameter space and classified into two groups based on their corresponding objective values computed using our SMC tool. The reported global sensitivities were calculated as the Kolmogorov-Smirnov statistics of cumulative frequency curves associated with the two groups.

## Electronic supplementary material

Supplementary Information
