# Supplementary material for: Quantitative assessment of cell fate decision between autophagy and apoptosis
Source: Sci Rep. 2017 Dec 14;7:17605. doi: 10.1038/s41598-017-18001-w (PMC5730598; doi:10.1038/s41598-017-18001-w)
Supplement: Supplementary file 1 — Supplementary Information [file 41598_2017_18001_MOESM1_ESM.pdf]

Supplementary Information

Quantitative assessment of cell fate decision between autophagy and apoptosis

B. Liu[1,*], Z. N. Oltvai[1,2], H. Bayir[3], G. A. Silverman[4], S. C. Pak[5], D. H. Perlmutter[4], I. Bahar[1,*]

Departments of [1]Computational & Systems Biology, [2]Pathology, [3]Critical Care, [5]Pediatrics, School of Medicine, University of Pittsburgh, Pittsburgh, PA 15213, USA.
[4]Department of Pediatrics, Washington University School of Medicine, St Louis, Missouri, MO 63110, USA.

SI Methods

ODE based Modeling

We describe the dynamics of our biochemical network using a system of ordinary differential equations (ODEs). For each molecular species x_i taking part in the pathway there will be an equation of the form $\frac{dx_i}{dt} = f_i(\mathbf{x}, \mathbf{c})$. Here f_i describes the kinetics of the reactions that produce and consume x_i, $\mathbf{x}$ are the molecular species taking part in these reactions while the vector of $\mathbf{c}$ gives the rate constants governing these reactions. We assume that the kinetic laws governing the individual reactions in the pathway are mass action law. Given that $\{x_1, x_2, \ldots, x_n\}$ is the set of variables and $\{c_1, c_2, \ldots, c_m\}$ the set of rate constants. Then each f_i will be of the form $f_i = \sum_{j=1}^{r_i} d_j n_{ij} g_j$, where r_i is the number of reactions associated with species x_i and $d_j = -1$ (or $d_j = +1$) if x_i is a reactant (or product) of the jth reaction. Further, $n_{ij} \in \mathbb{Z}$ denote the stoichiometric coefficients while g_j are rational functions of the form $g_j = c_\alpha x_a x_b$ (mass action) with $a, b \in \{1, 2, \ldots, n\}$ and $\alpha \in \{1, 2, \ldots, m\}$. Each x_i is real-valued function of t with $t \in \mathbb{R}_+$, where $\mathbb{R}_+$ denotes the set of non-negative real numbers. We realistically assume that $x_i(t)$ takes values in the interval $[L_i, U_i]$ where L_i and U_i are non-negative rationals with $L_i < U_i$. Hence the state space of the system will be $\mathbf{V} = [L_1, U_1] \times [L_2, U_2] \ldots \times [L_n, U_n] \subseteq \mathbb{R}_+^n$. Thus $\mathbf{V}$ will be a bounded subset of $\mathbb{R}_+^n$.

To capture the cell-to-cell variability and uncertainties regarding the initial states we define for each variable x_i an interval $[L_i^{init}, U_i^{init}]$ with $L_i \leq L_i^{init} < U_i^{init} \leq U_i$. We set $INIT = [L_1^{init}, U_1^{init}] \times [L_2^{init}, U_2^{init}] \ldots \times [L_n^{init}, U_n^{init}]$. The vector form of our system of ODEs is: $d\mathbf{x}/dt = F(\mathbf{x})$ with $\mathbf{x} = (x_1, x_2, \ldots, x_n)$ and $F(\mathbf{x}(i)) = f_i$. We define the flow $\Phi : \mathbb{R}_+ \times \mathbf{V} \to \mathbf{V}$ for arbitrary initial vectors $\mathbf{v}$. Intuitively, $\Phi(t, \mathbf{v})$ is the state reached under the ODE dynamics if the system starts at $\mathbf{v}$ at time 0. The dynamics will be of interest only up to a maximal time point T. We define a *trajectory* starting from $\mathbf{v} \in \mathbf{V}$ denoted $\sigma_\mathbf{v}$ to be the (continuous) function $\sigma_\mathbf{v} : [0, T] \to \mathbf{V}$ satisfying: $\sigma_\mathbf{v}(t) = \Phi_t(\mathbf{v})$. The behavior of our dynamical system is the set of trajectories given by $BEH = \{\sigma_\mathbf{v} \mid \mathbf{v} \in INIT\}$.

Statistical Model Checking

We define a bounded linear time temporal logic (BLTL) as follows. An atomic proposition in our logic will be of the form (i, ℓ, u) with $L_i \leq \ell < u \leq U_i$. Such a proposition will be interpreted as "the current concentration level of x_i falls in the interval $[\ell, u]$". We fix a finite set of such atomic propositions $AP = \{A_1, \ldots\ldots A_k\}$. The formulas of BLTL are: (i) Every atomic proposition as well as the constants *true*, *false* are BLTL formulas. (ii) If ψ, ψ' are BLTL formulas then $\neg\psi$ and $\psi \vee \psi'$ are BLTL formulas. (iii) If ψ, ψ' are BLTL formulas and $t \leq T$ is a positive integer then $\psi \mathbf{U}^{\leq t} \psi'$ and $\psi \mathbf{U}^t \psi'$ are BLTL formulas. The derived propositional operators such as $\wedge$, $\supset$, $\equiv$ and the temporal operators $\mathbf{G}^{\leq t}$, $\mathbf{G}^t$, $\mathbf{F}^{\leq t}$ and $\mathbf{F}^t$ are defined in the usual way. We will interpret the formulas of our logic at the finite set of time points $\mathcal{T} = \{0, 1, \ldots, T\}$. We assume T has been chosen such that it exceeds the last time for which experimental data is available. The semantics of the logic is defined in terms of the relation $\sigma, t \models \varphi$ where σ is a trajectory in BEH and $t \in \mathcal{T}$.

- $\sigma, t \models (i, \ell, u)$ iff $\ell \leq \sigma(t)(i) \leq u$ where $\sigma(t)(i)$ is the ith component of the n-dimensional vector $\sigma(t) \in \mathbf{V}$.
- $\neg$ and $\vee$ are interpreted in the usual way.
- $\sigma, t \models \psi \mathbf{U}^{\leq k} \psi'$ iff there exists k' such that $k' \leq k,\ t + k' \leq T$ and $\sigma, t + k' \models \psi'$. Further, $\sigma, t + k'' \models \psi$ for every $0 \leq k'' < k'$.
- $\sigma, t \models \psi \mathbf{U}^k \psi'$ iff $t + k \leq T$ and $\sigma, t + k \models \psi'$. Further, $\sigma, t + k' \models \psi$ for every $0 \leq k' < k$.

We define $models(\psi) = \{\sigma \mid \sigma, 0 \models \psi,\ \sigma \in BEH\}$. We shall make statements of the form $P_{>r}(\psi)$, where the intended meaning is that the probability that a trajectory in BEH belongs to $models(\psi)$ exceeds r. To assign precise meaning to such a statement, we define a probability measure over sets of trajectories. We identify BEH with $INIT$, and define the set $Models(\psi) \subseteq INIT$ as: $\mathbf{v} \in Models(\psi)$ iff there exists $\sigma \in models(\psi)$ with $\sigma(0) = \mathbf{v}$. To assign a probability to $Models(\psi)$, we construct a probability measure over the standard σ-algebra $\mathcal{B}$ generated by the open intervals contained in $INIT$. Suppose $\prod_{i=1}^n (\ell_i, u_i) \in \mathcal{B}$. We define $P(\prod_{i=1}^n (\ell_i, u_i)) = \prod_{i=1}^n \frac{(u_i - \ell_i)}{(U_i^{init} - L_i^{init})}$. It is a standard fact that P extends in a unique way to the probability measure $P : \mathcal{B} \to [0, 1]$ such that $P(INIT) = 1$ and $P(\emptyset) = 0$. We define the formulas as $P_{\geq r}\psi$ and $P_{\leq r'}\psi$ are probabilistic BLTL formulas provided $r \in [0, 1)$, $r' \in (0, 1]$ and ψ is a BLTL formula. We shall say that $\mathcal{S}$, the system of ODEs meets the specification $P_{\geq r}\psi$ – and this is denoted $\mathcal{S} \models P_{\geq r}\psi$ – iff $P(Models(\psi)) \geq r$, while $\mathcal{S} \models P_{\leq r'}\psi$ iff $P(Models(\psi)) \leq r'$.

We formulate whether $\mathcal{S} \models P_{\geq r}\psi$, as a sequential hypothesis test between the null hypothesis $\mathrm{H0}: p \geq r + \delta$ and the alternative hypothesis $\mathrm{H1}: p \leq r - \delta$, where $p = P(Models(\psi))$. Here, δ signifies the indifference region supplied by the user. The *strength* of the test is decided by parameters α and β which bound the Type-I (false positive) and Type-II (false negative) errors respectively. Thus the verification is carried out approximately but with guaranteed confidence levels and error bounds. The test proceeds by generating a sequence of sample trajectories $\sigma_1, \sigma_2, \ldots$ by randomly sampling an initial state from $INIT$. One assumes a corresponding sequence of Bernoulli random variables $y_1, y_2 \ldots$, where each y_k is assigned the value 1 if $\sigma_k, 0 \models \psi$. Otherwise y_k is assigned the value 0. For each $m \geq 1$, after drawing m samples, we compute a quantity q_m as:

$$q_m = \frac{[r-\delta]^{(\sum_{i=1}^{m} y_i)}[1-[r-\delta]]^{(m-\sum_{i=1}^{m} y_i)}}{[r+\delta]^{(\sum_{i=1}^{m} y_i)}[1-[r+\delta]]^{(m-\sum_{i=1}^{m} y_i)}}$$

Hypothesis H0 is accepted if $q_m \geq \widehat{A}$, and Hypothesis H1 is accepted if $q_m \leq \widehat{B}$. If neither is the case then another sample is drawn. The constants $\widehat{A}$ and $\widehat{B}$ are chosen such that it results in a test of strength (α, β). In practice, a good approximation is $\widehat{A} = \frac{1-\beta}{\alpha}$ and $\widehat{B} = \frac{\beta}{1-\alpha}$.

Parameter Estimation using Statistical Model Checking

We encode experimental data as a BLTL formula ψ_{exp}. Let $O \subseteq \{x_1, x_2, \ldots, x_n\}$ be the set of variables for which experimental data is available and which has been fixed as training data to be used for parameter estimation. Assume $\mathcal{T}_i = \{\tau_1^i, \tau_2^i, \ldots, \tau_{T_i}^i\}$ are the time points at which the concentration level of x_i has been measured and reported as $[\ell_t^i, u_t^i]$ for each $t \in \mathcal{T}_i$. Here the interval $[\ell_t^i, u_t^i]$ is so chosen that it reflects the noisiness, the limited precision and the cell-population-based nature of the experimental data. For each $t \in \mathcal{T}_i$ we define the formula $\psi_i^t = \mathbf{F}^t(i, \ell_t^i, u_t^i)$. Then $\psi_{exp}^i = \bigwedge_{t \in \mathcal{T}_i} \psi_i^t$. We then set $\psi_{exp} = \bigwedge_{i \in O} \psi_{exp}^i$.

We encode qualitative dynamic constraints as a BLTL formula ψ_{qlty}. We fix the probabilistic BLTL formula $P_{\geq r}(\psi_{exp} \wedge \psi_{qlty})$, where r will capture the confidence level with which we wish to assess the goodness of the fit of the current set of parameters to experimental data and qualitative trends. We also fix an indifference region δ and the strength of the test (α, β). We use the constants $r = 0.9$, $\delta = 0.05$, $\alpha = 0.05$ and $\beta = 0.05$.

Let $\theta = \{c_1, c_2, \ldots, c_K\}$ be the set of unknown rate constants whose values we wish to estimate. The outer loop of our parameter estimation procedure will run as follows. We shall assume for convenience that the search strategy uses a single set of parameter values (one for each unknown rate constant) in each round.

(i) Fix θ_0, which assigns a value to each unknown rate constant. This represents the initial guess. Set $\ell = 0$.

(ii) With θ_ℓ as the current set of rate constant values, run the statistical model checking procedure to verify the individual conjuncts of $\psi_{exp} \wedge \psi_{qlty}$ with the chosen strengths.

(iii) Based on the answers returned by these tests compute $F(\theta_\ell)$, where F is the objective function.

(iv) Check if the value of the objective function is sufficiently high or ℓ has reached a predetermined bound.

(v) If yes, return θ_ℓ as the estimated value.

(vi) Else fix a new set of rate constant values $\theta_{\ell+1}$ as dictated by the search strategy. Increment ℓ to $\ell + 1$ and return to step (ii).

The objective function is formed as follows. Let θ be an assignment of values to the unknown rate constants. Let J_{exp}^i $(= T_i)$ be the number of conjuncts in ψ_{exp} and J_{qlty} the number of conjuncts in ψ_{qlty}. Let $J_{exp}^{i,+}(\theta)$ be the number of formulas of the form ψ_i^t (a conjunct in ψ_{exp}^i) such that the statistical test for $P_{\geq r}(\psi_i^t)$ accepts the null hypothesis (that is, $P_{\geq r}(\psi_i^t)$ holds) with the strength $(\frac{\alpha}{J}, \beta)$, where $J = \sum_{i \in O} J_{exp}^i$. Similarly, let $J_{qlty}^+(\theta)$ be the number of conjuncts in ψ_{qlty} of the form $\psi_{\ell,qlty}$ that pass the statistical test $P_{\geq r}(\psi_{\ell,qlty})$ with the strength $(\frac{\alpha}{J}, \beta)$. Then $F(\theta)$ is computed via:

$$F(\theta) = J_{qlty}^+(\theta) + \sum_{i \in O} \frac{J_{exp}^{i,+}}{J_{exp}^i} \tag{1}$$

Thus the goodness to fit of θ is measured by how well it agrees with the qualitative properties as well as the number of experimental data points with which there is acceptable agreement. To avoid over-training the model, we do not insist that every qualitative property and every data point must fit well with the dynamics predicted by θ.

The search strategy deployed in step (vi) above will use the values $F(\theta_\ell)$ to traverse the space of candidate parameter vectors. We employ a global method called Stochastic Ranking Evolutionary Strategy (SRES) [2], since it is known to perform well in the context of pathway models [1].

Table S1: List of components

Name	Description
JNK	Inactive c-Jun N-terminal kinases
JNK*	Active c-Jun N-terminal kinases
DAPK	Inactive Death-associated protein kinase
DAPK*	Active Death-associated protein kinase
AMPK	Inactive Death-associated protein kinase
AMPK*	Active Death-associated protein kinase
cAMP	Cyclic adenosine monophosphate
RHEB	Inactive Ras Homolog Enriched In Brain
RHEB*	Active Ras Homolog Enriched In Brain
mTORC1	Inactive mechanistic target of rapamycin complex 1
mTORC1*	Active mechanistic target of rapamycin complex 1
ULK1	Inactive Unc-51 like autophagy activating kinase 1
ULK1*	Active Unc-51 like autophagy activating kinase 1
TFEBi	Inactive Transcription Factor EB
TFEB	Active Transcription Factor EB
PLCϵ	Inactive Phospholipase C, epsilon 1
PLCϵ*	Active Phospholipase C, epsilon 1
IP_3	Inositol trisphosphate
IP_2	Inositol bisphosphate
IP	Inositol monophosphate
Ins	Inositol
PIP_2	Phosphatidylinositol 4,5-bisphosphate
IP_3R	Inositol 1,4,5-trisphosphate receptor
Ca^{2+} (IC)	Cytoplasmic calcium
Ca^{2+} (EC)	Extracellular calcium
Ca^{2+} (ER)	Calcium in endoplasmic reticulum
Calpain	Inactive calpain
Calpain*	Active calpain
Beclin1	A mammalian ortholog of the yeast autophagy-related gene (Atg) 6
Beclin1C	C-terminal fragment of Beclin1
UVRAG	UV radiation resistance-associated gene protein
BCL2	Active anti-apoptotic Bcl-2 family proteins
BCL2i	Inactive anti-apoptotic Bcl-2 family proteins
p53c	p53 in cytoplasm
p53m	p53 in mitochondria

Continued on next page

Table S1 – continued from previous page

Name	Description
p53*	stress-induced p53 for apoptotic activation
DRAM	Damage-regulated autophagy modulator
Mdm2	Mdm2/E3 ligase proteins
Bax	Pro-apoptotic Bcl-2 family member
Bax*	Oligomerized pro-apoptotic Bcl-2 family proteins
Bid	BH3-only pro-apoptotic proteins
tBid	Truncated BH3-only pro-apoptotic proteins
cyt c	cytochrome c in cytoplasm
caspase	procaspases
caspase*	Activated caspases
TSC1/2	Inactive tuberous sclerosis proteins 1 and 2
TSC1/2*	Active tuberous sclerosis proteins 1 and 2
GPCR*	Activated G-protein-coupled receptors
Gα, β, γ	G protein subunits α, β, and γ
Gα	G protein subunit α
Gβ, γ	G protein subunit β and γ
Gα*	Activated G protein subunit α
AC	Adenylyl cyclase
EPAC	Inactive exchange protein directly activated by cAMP
EPAC*	Active exchange protein directly activated by cAMP
SERCA	Sarco/endoplasmic reticulum Ca^{2+}-ATPase
CaMKKβ	Inactive calcium/calmodulin-dependent protein kinase kinase 2, beta
CaMKKβ*	Active calcium/calmodulin-dependent protein kinase kinase 2, beta
ATG5	Autophagy related 5 protien
ATG5t	Truncated ATG5
PI_3K	Inactive phosphoinositide 3-kinase
PI_3K*	Active phosphoinositide 3-kinase
AKT	Inactive protein kinase B
AKT*	Active protein kinase B
PKA	Inactive protein kinase A
PKA*	Active protein kinase A
PKC*	Active protein kinase C
MAPK15	Inactive mitogen-activated protein kinase 15
MAPK15*	Active mitogen-activated protein kinase 15
Insulin	Insulin

Continued on next page

Table S1 – continued from previous page

Name	Description
AC:Gα*	AC and active G protein subunit alpha complex
mTORC1*:ULK1*	Active mTORC1 and active ULK1 complex
mTORC1*:ULK1	Active mTORC1 and inactive ULK1 complex
Beclin1:UVRAG	Beclin1 and UVRAG complex
IP_3R:IP_3	IP_3R and IP_3 complex
BCL2:IP_3R	IP_3R and Bcl2 complex
Beclin1:BCL2	Beclin1 and Bcl2 complex
BCL2:ATG5t	ATG5t and Bcl2 complex
BCL2:Bax	Bax and Bcl2 complex
p53m:BCL2	p53m and Bcl2 complex
UVRAG:Bax	UVRAG and Bax complex
JUN	Inactive AP-1 transcription factor subunit
JUN*	Active AP-1 transcription factor subunit
PERK	Inactive protein kinase R (PKR)-like endoplasmic reticulum kinase
PERK*	Active protein kinase R (PKR)-like endoplasmic reticulum kinase
ATF4	Inactive activating transcription factor 4
ATF4*	Active activating transcription factor 4
PUMA	p53 upregulated modulator of apoptosis
PUMA:BCL2	PUMA and BCL2 complex
phagophore	Isolation membrane
autophagosome	A spherical structure with double layer membranes formed for engulfing and delivering to the lysosomes the cell components/organelles eliminated by autophagy
NStress	Nutritional Stress
EStress	ER Stress
DStress	Stress caused by DNA damage

Table S2: Reactions and parameters values.

Number	Reaction	Parameters
1	AMPK + NStress $\xrightarrow{k_1}$ AMPK* + NStress	$k_1 = 0.000164$ nM^{-1}min^{-1}
2	AMPK* $\xrightarrow{k_2}$ AMPK	$k_2 = 0.0159$ min^{-1}
3	AMPK* + mTORC1* $\xrightarrow{k_3}$ AMPK* + mTORC1	$k_3 = 0.000167$ nM^{-1}min^{-1}
4	AMPK* + mTORC1*:ULK1* $\xrightarrow{k_4}$ AMPK* + mTORC1 + ULK*	$k_4 = 0.00017$ nM^{-1}min^{-1}
5	mTORC1* $\xrightarrow{k_5}$ mTORC1	$k_5 = 0.01682$ min^{-1}
6	ULK + mTORC1* $\underset{k_7}{\overset{k_6}{\rightleftharpoons}}$ mTORC1*:ULK	$k_6 = 0.000159$ nM^{-1}min^{-1}, $k_7 = 0.0168$ min^{-1}
7	ULK* + mTORC1* $\underset{k_9}{\overset{k_8}{\rightleftharpoons}}$ mTORC1*:ULK	$k_8 = 0.000158$ nM^{-1}min^{-1}, $k_9 = 0.0165$ min^{-1}
8	AMPK* + ULK1 $\xrightarrow{k_{10}}$ AMPK* + ULK*	$k_{10} = 0.000166$ nM^{-1}min^{-1}
9	ULK* $\xrightarrow{k_{11}}$ ULK* + phagophore	$k_{11} = 0.0164$ min^{-1}
10	AMPK* + TSC1/2 $\xrightarrow{k_{12}}$ AMPK* + TSC1/2*	$k_{12} = 0.000171$ nM^{-1}min^{-1}
11	AKT* + TSC1/2* $\xrightarrow{k_{13}}$ AKT* + TSC1/2	$k_{13} = 0.0162$ nM^{-1}min^{-1}
12	RHEB* + TSC1/2* $\xrightarrow{k_{14}}$ RHEB + TSC1/2*	$k_{14} = 0.00169$ nM^{-1}min^{-1}
13	RHEB* + mTORC1 $\xrightarrow{k_{15}}$ RHEB* + mTORC1*	$k_{15} = 0.00017$ nM^{-1}min^{-1}
14	RHEB $\xrightarrow{k_{16}}$ RHEB*	$k_{16} = 0.015$ min^{-1}
15	ULK1* $\xrightarrow{k_{17}}$ ULK1	$k_{17} = 0.00169$ min^{-1}
16	AMPK* + ULK1* $\xrightarrow{k_{18}}$ AMPK + ULK*	$k_{18} = 0.000163$ nM^{-1}min^{-1}
17	phagophore + Beclin1 $\xrightarrow{k_{19}}$ preautophagosome + Beclin1	$k_{19} = 0.000033$ nM^{-1}min^{-1}
18	phagophore + Beclin1:UVRAG $\xrightarrow{k_{20}}$ preautophagosome + Beclin1:UVRAG	$k_{20} = 0.000166$ nM^{-1}min^{-1}
19	Beclin1 + UVRAG $\underset{k_{22}}{\overset{k_{21}}{\rightleftharpoons}}$ Beclin1:UVRAG	$k_{21} = 0.000165$ nM^{-1}min^{-1}, $k_{22} = 0.0168$ min^{-1}
20	$\xrightarrow{k_{23}}$ Beclin1	$k_{23} = 0.000185$ nM^{-1}
21	Beclin1C $\xrightarrow{k_{24}}$ cyt c + Beclin1C	$k_{24} = 0.000035$ min^{-1}
22	Beclin1C $\xrightarrow{k_{25}}$ ϕ	$k_{25} = 0.082$ min^{-1}
23	phagophore $\xrightarrow{k_{26}}$ ϕ	$k_{26} = 0.00168$ min^{-1}
24	ATG5 + phagophore $\xrightarrow{k_{27}}$ ATG5 + autophagosome	$k_{27} = 0.0166$ nM^{-1}min^{-1}
25	autophagosome $\xrightarrow{k_{28}}$ ϕ	$k_{28} = 0.0167$ min^{-1}
26	autopahgosome + NStress $\xrightarrow{k_{29}}$ autopahgosome	$k_{29} = 0.017$ nM^{-1}min^{-1}
27	autopahgosome + NStress $\xrightarrow{k_{29}}$ autopahgosome	$k_{29} = 0.017$ nM^{-1}min^{-1}
28	autopahgosome + EStress $\xrightarrow{k_{29}}$ autopahgosome	$k_{29} = 0.017$ nM^{-1}min^{-1}
29	$\xrightarrow{k_{30}}$ phagophore	$k_{30} = 0.0000017$ nM^{-1}
30	EStress + PERK $\xrightarrow{k_{30}}$ PERK*	$k_{30} = 0.000002$ nM^{-1}min^{-1}
31	ATF* $\xrightarrow{k_{31}}$ phagophore	$k_{32} = 0.00016$ min^{-1}
32	PERK* + ATF4 $\xrightarrow{k_{32}}$ PERK + ATF4*	$k_{32} = 0.014$ nM^{-1}min^{-1}
33	preautophagophore $\xrightarrow{k_{33}}$ ϕ	$k_{33} = 0.00165$ min^{-1}
34	DStress + DAPK $\xrightarrow{k_{34}}$ DStress + DAPK*	$k_{34} = 0.00062$ nM^{-1}min^{-1}

Continued on next page

Number	Reaction	Parameters
35	DAPK* $\xrightarrow{k_{35}}$ DAPK	k_{35} = 0.0178 min^{-1}
36	EPAC* + PLCϵ $\xrightarrow{k_{36}}$ EPAC* + PLCϵ*	k_{36} = 0.0152 nM^{-1}min^{-1}
37	PLCϵ* $\xrightarrow{k_{37}}$ PLCϵ	k_{37} = 0.000193 min^{-1}
38	PLCϵ* + PIP$_2$ $\xrightarrow{k_{38}}$ PLCϵ* + IP$_3$	k_{38} = 0.02 nM^{-1}min^{-1}
39	Ca^{2+}(ER) + IP$_3$R:IP$_3$ $\xrightarrow{k_{39}}$ Ca^{2+}(IC) + IP$_3$R:IP$_3$	k_{39} = 0.0159 nM^{-1}min^{-1}
40	Ca^{2+}(IC) + SERCA $\xrightarrow{k_{40}}$ Ca^{2+}(ER) + SERCA	k_{40} = 0.00062 nM^{-1}min^{-1}
41	Ca^{2+}(IC) + Bax $\xrightarrow{k_{41}}$ Ca^{2+}(IC) + Bax*	k_{41} = 0.00015 nM^{-1}min^{-1}
42	IP$_3$R + IP$_3$ $\underset{k_{43}}{\overset{k_{42}}{\rightleftharpoons}}$ IP$_3$R:IP$_3$	k_{42} = 0.000169 nM^{-1}min^{-1}, k_{43} = 0.0154 min^{-1}
43	IP$_3$ $\xrightarrow{k_{44}}$ IP$_2$	k_{44} = 0.017 min^{-1}
44	IP$_2$ $\xrightarrow{k_{45}}$ IP	k_{45} = 0.017 min^{-1}
45	IP $\xrightarrow{k_{46}}$ Ins	k_{46} = 0.017 min^{-1}
46	Ins $\xrightarrow{k_{47}}$ PIP$_2$	k_{47} = 0.017 min^{-1}
47	IP$_3$R:IP$_3$ + phagophore $\xrightarrow{k_{48}}$ IP$_3$R:IP$_3$	k_{48} = 0.00014 nM^{-1}min^{-1}
48	Ca^{2+}(IC) + CaMKKβ $\xrightarrow{k_{49}}$ Ca^{2+}(IC) + CaMKKβ*	k_{49} = 0.0000167 nM^{-1}min^{-1}
49	CaMKKβ* $\xrightarrow{k_{50}}$ Ca^{2+}(IC) + CaMKKβ	k_{50} = 0.0166 min^{-1}
50	AMPK + CaMKKβ* $\xrightarrow{k_{51}}$ AMPK* + CaMKKβ*	k_{51} = 0.000165 nM^{-1}min^{-1}
51	IP$_3$R + BCL2 $\underset{k_{53}}{\overset{k_{52}}{\rightleftharpoons}}$ IP$_3$R:BCL2	k_{52} = 0.000177 nM^{-1}min^{-1}, k_{53} = 0.0152min^{-1}
52	Beclin1 + BCL2 $\underset{k_{55}}{\overset{k_{54}}{\rightleftharpoons}}$ Beclin1:BCL2	k_{54} = 0.00017 nM^{-1}min^{-1}, k_{55} = 0.0154 min^{-1}
53	BCL2p $\xrightarrow{k_{56}}$ BCL2	k_{56} = 0.015 min^{-1}
54	Ca^{2+}(IC) + Calpain $\xrightarrow{k_{57}}$ Ca^{2+}(IC) + Calpain*	k_{57} = 0.000188 nM^{-1}min^{-1}
55	Calpain* $\xrightarrow{k_{58}}$ Calpain	k_{58} = 0.016 min^{-1}
56	Calpain* + Gα $\xrightarrow{k_{59}}$ Calpain* + Gα*	k_{59} = 0.000193 nM^{-1}min^{-1}
57	Calpain* + ATG5 $\xrightarrow{k_{60}}$ Calpain* + ATG5t	k_{60} = 0.0002 nM^{-1}min^{-1}
58	ATG5t + BCL2 $\underset{k_{62}}{\overset{k_{61}}{\rightleftharpoons}}$ ATG5:BCL2	k_{61} = 0.00019 nM^{-1}min^{-1}, k_{62} = 0.0000047 min^{-1}
59	Calpain* + Bid $\xrightarrow{k_{63}}$ Calpain* + tBid	k_{63} = 0.000168 nM^{-1}min^{-1}
60	Calpain* + Beclin1 $\xrightarrow{k_{64}}$ Calpain*	k_{64} = 0.000056 nM^{-1}min^{-1}
61	AC:Gα* $\xrightarrow{k_{65}}$ AC:Gα* + cAMP	k_{65} = 0.02 min^{-1}
62	cAMP $\xrightarrow{k_{66}}$ ϕ	k_{66} = 0.0833 min^{-1}
63	cAMP + EPAC $\xrightarrow{k_{67}}$ cAMP + EPAC*	k_{67} = 0.00021 nM^{-1}min^{-1}
64	EPAC* $\xrightarrow{k_{68}}$ EPAC	k_{68} = 0.0188 nM^{-1}min^{-1}
65	G* $\xrightarrow{k_{69}}$ G	k_{69} = 0.0166 min^{-1}
66	AC + Gα $\underset{k_{71}}{\overset{k_{70}}{\rightleftharpoons}}$ AC:Gα	k_{70} = 0.002 nM^{-1}min^{-1}, k_{71} = 0.0195 min^{-1}
67	Gα,β,γ + GPCR* $\xrightarrow{k_{72}}$ Gα + Gβ,γ + GPCR*	k_{72} = 0.021 min^{-1}
68	Gα + Gβ,γ $\xrightarrow{k_{73}}$ Gα,β,γ	k_{73} = 0.00003 nM^{-1}min^{-1}

Continued on next page

Table S2 – continued from previous page

Number	Reaction	Parameters
69	BCL2 $+$ Bax $\underset{k_{75}}{\overset{k_{74}}{\rightleftharpoons}}$ BCL2:Bax	$k_{74} = 0.00021$ nM^{-1}min^{-1}, $k_{75} = 0.019$ min^{-1}
70	Bax $+$ p53m $\xrightarrow{k_{76}}$ Bax* $+$ p53m	$k_{76} = 0.0001667$ nM^{-1}min^{-1}
71	Bax* $\xrightarrow{k_{77}}$ Bax	$k_{77} = 0.084$ min^{-1}
72	Bax $\xrightarrow{k_{78}}$ ϕ	$k_{78} = 0.017$ min^{-1}
73	UVRAG $+$ Bax $\underset{k_{80}}{\overset{k_{79}}{\rightleftharpoons}}$ UVRAG:Bax	$k_{79} = 0.000156$ nM^{-1}min^{-1}, $k_{80} = 0.00154$ min^{-1}
74	cyt$c$ $+$ casepase $\xrightarrow{k_{81}}$ cyt$c$ $+$ caspase*	$k_{81} = 0.000167$ nM^{-1}min^{-1}
75	Bid $+$ casepase* $\xrightarrow{k_{82}}$ tBid $+$ caspase*	$k_{82} = 0.000165$ nM^{-1}min^{-1}
76	tBid $\xrightarrow{k_{83}}$ ϕ	$k_{83} = 0.0153$ min^{-1}
77	$\xrightarrow{k_{84}}$ Bid	$k_{84} = 0.000155$ nM^{-1}
78	JNK* $+$ caspase $\xrightarrow{k_{85}}$ JNK* $+$ casepse*	$k_{85} = 0.00007$ nM^{-1}min^{-1}
79	DStress $\xrightarrow{k_{86}}$ DSress $+$ p53c	$k_{86} = 0.015$ min^{-1}
80	p53c $\xrightarrow{k_{87}}$	$k_{87} = 0.014$ min^{-1}
81	p53c $\underset{k_{89}}{\overset{k_{88}}{\rightleftharpoons}}$ p53*	$k_{88} = 0.016$ min^{-1}, $k_{89} = 0.018$ min^{-1}
82	p53* $\xrightarrow{k_{90}}$ p53* $+$ Mdm2	$k_{90} = 0.0142$ min^{-1}
83	p53c $+$ Mdm2 $\xrightarrow{k_{91}}$ p53m $+$ Mdm2	$k_{91} = 0.000158$ nM^{-1}min^{-1}
84	p53* $\xrightarrow{k_{92}}$ p53* $+$ Bax	$k_{92} = 0.0164$ min^{-1}
85	p53m $+$ BCL2 $\underset{k_{94}}{\overset{k_{93}}{\rightleftharpoons}}$ p53:BCL2	$k_{93} = 0.000162$ nM^{-1}min^{-1}, $k_{94} = 0.00001$ min^{-1}
86	Mdm2 $\xrightarrow{k_{95}}$ ϕ	$k_{95} = 0.018$ min^{-1}
87	p53* $\xrightarrow{k_{96}}$ p53* $+$ DRAM	$k_{96} = 0.018$ min^{-1}
88	DRAM $\xrightarrow{k_{97}}$	$k_{97} = 0.0162$ min^{-1}
89	DRAM $+$ preautophagosome $\xrightarrow{k_{98}}$ DRAM $+$ autophagosome*	$k_{98} = 0.000166$ nM^{-1}min^{-1}
90	p53c $+$ AMPK* $\xrightarrow{k_{99}}$ p53c $+$ AMPK	$k_{99} = 0.000157$ nM^{-1}min^{-1}
91	p53* $\xrightarrow{k_{100}}$ p53* $+$ PUMA	$k_{100} = 0.0085$ min^{-1}
92	PUMA $+$ BCL2 $\underset{k_{102}}{\overset{k_{101}}{\rightleftharpoons}}$ PUMA:BCL2	$k_{101} = 0.001$ nM^{-1}min^{-1}, $k_{102} = 0.05$ min^{-1}
93	PUMA $\xrightarrow{k_{103}}$ ϕ	$k_{103} = 0.0072$ min^{-1}
94	Bax* $\xrightarrow{k_{104}}$ cyt$c$ $+$ Bax*	$k_{104} = 0.0165$ min^{-1}
95	cyt c $+$ casepase $\xrightarrow{k_{105}}$ cyt c $+$ caspase*	$k_{105} = 0.0017$ nM^{-1}min^{-1}
96	cyt c $\xrightarrow{k_{106}}$	$k_{106} = 0.0166$ min^{-1}
97	mTORC1* $+$ TFEBi $\xrightarrow{k_{107}}$ mTORC1* $+$ TFEB	$k_{107} = 0.00017$ nM^{-1}min^{-1}
98	phagophore $+$ TFEB $\xrightarrow{k_{108}}$ autophagosome $+$ TFEB	$k_{108} = 0.0000016$ nM^{-1}min^{-1}
99	TFEB $\xrightarrow{k_{109}}$ TFEBi	$k_{109} = 0.00017$ min^{-1}
100	Insulin $+$ PI3K $\xrightarrow{k_{110}}$ Insulin $+$ PI3K*	$k_{110} = 0.024$ nM^{-1}min^{-1}
101	PI3K* $\xrightarrow{k_{111}}$ PI3K	$k_{111} = 0.022$ min^{-1}
102	AKT $+$ PI3K* $\xrightarrow{k_{112}}$ AKT* $+$ PI3K	$k_{112} = 0.0189$ nM^{-1}min^{-1}

Continued on next page

Table S2 – continued from previous page

Number	Reaction	Parameters
103	AKT* $\xrightarrow{k_{113}}$ AKT	$k_{113} = 0.0176$ min^{-1}
104	AKT* + TSC1/2* $\xrightarrow{k_{114}}$ AKT* + TSC1/2	$k_{114} = 0.0155$ nM^{-1}min^{-1}
105	AKT* + mTORC1*:ULK1* $\xrightarrow{k_{115}}$ AKT* + mTORC1] + ULK*]	$k_{115} = 0.000161$ nM^{-1}min^{-1}
106	JNK* + BCL2 $\xrightarrow{k_{116}}$ JNK* + BCL2p	$k_{116} = 0.000172$ nM^{-1}min^{-1}
107	JNK* + Beclin1:BCL2 $\xrightarrow{k_{117}}$ JNK* + Beclin1 + BCL2p	$k_{117} = 0.000169$ nM^{-1}min^{-1}
108	DAPK* + Beclin1:BCL2 $\xrightarrow{k_{118}}$ DAPK* + Beclin1 + BCL2	$k_{118} = 0.031$ nM^{-1}min^{-1}
109	EStress + JNK $\xrightarrow{k_{119}}$ EStress + JNK*	$k_{119} = 0.000161$ nM^{-1}min^{-1}
110	JNK* $\xrightarrow{k_{120}}$ JNK	$k_{120} = 0.0159$ min^{-1}
111	NStress + MAPK15 $\xrightarrow{k_{121}}$ NStress + MAPK15	$k_{121} = 0.0034$ nM^{-1}min^{-1}
112	MAPK15* $\xrightarrow{k_{122}}$ MAPK15	$k_{122} = 0.087$ min^{-1}
113	PKA + MAPK15* $\xrightarrow{k_{123}}$ PKA* + MAPK15*	$k_{123} = 0.00052$ nM^{-1}min^{-1}
114	PKA* $\xrightarrow{k_{124}}$ PKA	$k_{124} = 0.078$ min^{-1}
115	PKA* + phagophore $\xrightarrow{k_{125}}$ PKA* + preautophagosome	$k_{125} = 0.000026$ nM^{-1}min^{-1}
116	PKC* + preautophagosome $\xrightarrow{k_{126}}$ PKA* + autophagosome	$k_{126} = 0.000053$ nM^{-1}min^{-1}
117	$\xrightarrow{k_{83}}$ ATG5	$k_{127} = 0.017$ min^{-1}
118	ATG5t $\xrightarrow{k_{83}}$	$k_{128} = 0.016$ min^{-1}
119	ATG5 $\xrightarrow{k_{83}}$	$k_{129} = 0.00169$ min^{-1}

Table S3: List of parameters and their ranges.

Parameter	Value Ranges	Parameter	Value Ranges	Parameter	Value Ranges	Parameter	Value Ranges
k_1	[0, 0.1]	k_2	[0, 1]	k_3	[0, 0.1]	k_4	[0, 0.1]
k_5	[0, 1]	k_6	[0, 0.1]	k_7	[0, 1]	k_8	[0, 1]
k_9	[0, 0.1]	k_{10}	[0, 0.1]	k_{11}	[0, 1]	k_{12}	[0, 0.1]
k_{13}	[0, 0.1]	k_{14}	[0, 1]	k_{15}	[0, 0.1]	k_{16}	[0, 1]
k_{17}	[0, 0.1]	k_{18}	[0, 0.01]	k_{19}	[0, 0.1]	k_{20}	[0, 1]
k_{21}	[0, 0.1]	k_{22}	[0, 1]	k_{23}	[0, 0.1]	k_{24}	[0, 0.1]
k_{25}	[0, 1]	k_{26}	[0, 1]	k_{27}	[0, 1]	k_{28}	[0, 1]
k_{29}	[0, 1]	k_{30}	[0, 1]	k_{31}	[0, 1]	k_{32}	[0, 0.1]
k_{33}	[0, 1]	k_{34}	[0, 1]	k_{35}	[0, 0.1]	k_{36}	[0, 0.1]
k_{37}	[0, 1]	k_{38}	[0, 1]	k_{39}	[0, 0.1]	k_{40}	[0, 1]
k_{41}	[0, 0.1]	k_{42}	[0, 1]	k_{43}	[0, 0.1]	k_{44}	[0, 1]
k_{45}	[0, 0.1]	k_{46}	[0, 1]	k_{47}	[0, 1]	k_{48}	[0, 1]
k_{49}	[0, 0.1]	k_{50}	[0, 0.01]	k_{51}	[0, 1]	k_{52}	[0, 0.1]
k_{53}	[0, 0.1]	k_{54}	[0, 1]	k_{55}	[0, 1]	k_{56}	[0, 1]
k_{57}	[0, 0.1]	k_{58}	[0, 1]	k_{59}	[0, 0.1]	k_{60}	[0, 1]
k_{61}	[0, 1]	k_{62}	[0, 1]	k_{63}	[0, 1]	k_{64}	[0, 1]
k_{65}	[0, 1]	k_{66}	[0, 1]	k_{67}	[0, 1]	k_{68}	[0, 1]
k_{69}	[0, 1]	k_{70}	[0, 1]	k_{71}	[0, 0.1]	k_{72}	[0, 1]
k_{73}	[0, 1]	k_{74}	[0, 0.1]	k_{75}	[0, 0.1]	k_{76}	[0, 0.1]
k_{77}	[0, 0.1]	k_{78}	[0, 0.1]	k_{79}	[0, 0.1]	k_{80}	[0, 0.1]
k_{81}	[0, 0.01]	k_{82}	[0, 0.1]	k_{83}	[0, 1]	k_{84}	[0, 1]
k_{85}	[0, 0.1]	k_{86}	[0, 0.1]	k_{87}	[0, 1]	k_{88}	[0, 1]
k_{89}	[0, 0.01]	k_{90}	[0, 1]	k_{91}	[0, 1]	k_{92}	[0, 0.01]
k_{93}	[0, 1]	k_{94}	[0, 1]	k_{95}	[0, 0.01]	k_{96}	[0, 1]
k_{97}	[0, 1]	k_{98}	[0, 1]	k_{99}	[0, 0.01]	k_{100}	[0, 1]
k_{101}	[0, 1]	k_{102}	[0, 0.01]	k_{103}	[0, 0.01]	k_{104}	[0, 0.01]
k_{105}	[0, 1]	k_{106}	[0, 0.01]	k_{107}	[0, 0.01]	k_{108}	[0, 0.01]
k_{109}	[0, 0.1]	k_{110}	[0, 0.1]	k_{111}	[0, 0.1]	k_{112}	[0, 0.1]
k_{113}	[0, 0.1]	k_{114}	[0, 0.1]	k_{115}	[0, 0.1]	k_{116}	[0, 0.1]
k_{117}	[0, 0.1]	k_{118}	[0, 0.1]	k_{119}	[0, 0.1]	k_{120}	[0, 0.1]
k_{121}	[0, 0.1]	k_{122}	[0, 0.01]	k_{123}	[0, 0.01]	k_{124}	[0, 0.01]
k_{125}	[0, 0.1]	k_{126}	[0, 0.1]	k_{127}	[0, 0.1]	k_{128}	[0, 0.1]
k_{129}	[0, 0.01]						

References

[1] C. G. Moles, P. Mendes, and J. R. Banga. Parameter estimation in biochemical pathways: A comparison of global optimization methods. *Genome Res.*, 13(11):2467 –2474, 2003.

[2] T. Runarsson and X. Yao. Stochastic ranking for constrained evolutionary optimization. *IEEE T. Evolut. Comput.*, 4:284–294, 2000.
